# Metformin as a Disease-Modifying Agent in Autosomal Dominant Polycystic Kidney Disease: A Systematic Review of Preclinical and Clinical Evidence

**DOI:** 10.3390/cimb47090715

**Published:** 2025-09-03

**Authors:** Aleksandra Maciejczyk, Mariusz Niemczyk

**Affiliations:** 1 Faculty of Medicine, Medical University of Warsaw, 02-091 Warsaw, Poland; 2 Department of Transplantology, Immunology, Nephrology and Internal Diseases, Medical University of Warsaw, Nowogrodzka 59, 02-006 Warsaw, Poland

**Keywords:** metformin, autosomal dominant polycystic kidney disease, ADPKD, inflammation, CKD progression

## Abstract

Autosomal dominant polycystic kidney disease (ADPKD) is a common inherited kidney disorder marked by cyst growth and progressive renal failure. This systematic review aims to summarize current preclinical and clinical evidence on the potential role of metformin in ADPKD, focusing on its effects on glucose metabolism, kidney function, inflammation, and survival. A comprehensive search was conducted in PubMed and Google Scholar up to June 2025, following PRISMA guidelines. Forty-two articles met the inclusion criteria and were analyzed. Included studies examined metformin use in ADPKD patients or models and reported outcomes such as renal function, cyst growth, metabolic markers, and mortality. In preclinical studies, it reduced cyst formation, improved kidney structure, and decreased inflammation. Clinical studies confirmed its safety and suggested benefits in slowing kidney function decline, especially in early-stage ADPKD. Metformin may be a promising supportive therapy in ADPKD due to its metabolic and anti-inflammatory effects.

## 1. Introduction

Autosomal dominant polycystic kidney disease (ADPKD) is the most common inherited kidney disorder, affecting approximately 1 in 400 to 1000 individuals worldwide [1]. Characterized by progressive formation and enlargement of bilateral renal cysts, ADPKD leads to a gradual loss of kidney function and ultimately results in end-stage renal disease (ESRD) in nearly half of the affected patients by their sixth decade of life [2,3,4,5,6]. In the majority of patients, this condition is caused by pathogenic variants in the PKD1 and PKD2 genes [7,8].

Recent studies have highlighted the presence of glucose metabolism abnormalities in patients with ADPKD, which may play a role in disease progression. These findings have prompted a growing interest in repurposing metabolic agents to target both the systemic and renal-specific aspects of ADPKD pathophysiology. Among these, metformin—a widely used oral antihyperglycemic agent for type 2 diabetes mellitus (T2DM)—has emerged as a promising candidate. Metformin exerts its primary effects via activation of AMP-activated protein kinase (AMPK), a cellular energy sensor that modulates metabolic homeostasis [9].

Given the metabolic alterations observed in ADPKD and the mechanistic rationale for AMPK activation, this review aims to explore the potential therapeutic benefits of metformin in ADPKD. Unlike previous reviews, it integrates metabolic, immunological, and clinical aspects into a unified perspective, highlighting how mitochondrial modulation, energy metabolism, and signaling pathways converge in cyst growth. Moreover, it proposes future therapeutic directions by comparing metformin with other emerging agents, such as tolvaptan, thereby offering a broader translational context. Emphasis will be placed on its role in correcting glucose metabolism disturbances, its impact on disease progression and renal outcomes, and its potential to improve hard clinical endpoints such as renal failure and mortality.

## 2. Materials and Methods (PRISMA Guidelines)

**Figure 1:**
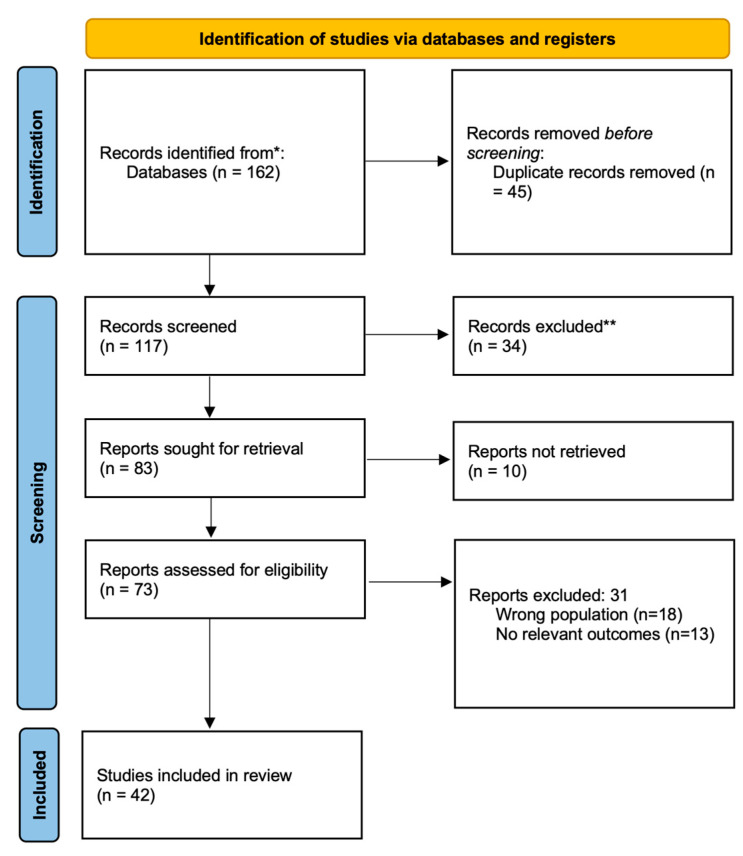
PRISMA 2020 flow diagram illustrating the process of study selection for inclusion in the systematic review. * PubMed, Google Scholar; ** editorials, letters to the editor, commentaries, conference abstracts without full data, studies not primarily focused on the topic.

This systematic review was conducted in accordance with the Preferred Reporting Items for Systematic Reviews and Meta-Analyses (PRISMA) 2020 guidelines [10], to ensure methodological transparency and reproducibility. The PRISMA flowchart summarizing the study selection process is presented in Figure 1. The review protocol was prospectively registered in the PROSPERO database (CRD420251062402). The objective of this review was to synthesize available preclinical and clinical evidence on the potential therapeutic benefits of metformin in ADPKD, with particular attention to its effects on glucose metabolism, renal function, mortality, inflammation, and other disease-related pathways.

To identify relevant studies, a comprehensive search of two major electronic databases—PubMed and Google Scholar—was performed up to June 2025. The search strategy employed a combination of controlled vocabulary and free-text terms, including: “metformin”, “AMPK activator”, “autosomal dominant polycystic kidney disease”, “ADPKD”, “renal function”, “eGFR”, “cyst growth”, and “mortality”. Boolean operators (“AND” and “OR”) were used to optimize the retrieval of relevant articles. In addition to database searching, the reference lists of included studies were manually screened to identify any additional eligible articles.

Studies were included if they involved individuals with ADPKD models of the disease, used metformin either alone or in combination with other drugs, and assessed relevant outcomes such as glucose metabolism, kidney function (e.g., eGFR, kidney volume), mortality, cyst burden, inflammatory markers, or systemic complications. Only articles published in English or Polish were considered. Eligible study types included randomized controlled trials, cohort studies (prospective and retrospective), case-control and observational studies, preclinical experiments (in vivo and in vitro), as well as literature reviews. Editorials, letters to the editor, commentaries, conference abstracts without full data, and studies not primarily focused on metformin use in ADPKD were excluded.

To ensure a systematic and transparent approach to study selection, the eligibility criteria were defined according to the PICO framework (Population, Intervention, Comparator, Outcome), commonly used in systematic reviews. Specifically, the population (P) included patients or animal models with autosomal dominant polycystic kidney disease (ADPKD); the intervention (I) was treatment with metformin; the comparator (C) was absence of metformin treatment; and the outcomes (O) included progression of renal function (e.g., eGFR decline), mortality, cyst burden, inflammation, or glucose metabolism.

For each clinical outcome, we assessed the certainty of evidence using the Grading of Recommendations Assessment, Development and Evaluation (GRADE) approach across five domains: risk of bias, inconsistency, indirectness, imprecision and publication bias. Randomized controlled trials were initially rated as high certainty and observational studies as low certainty, with subsequent downgrading or upgrading as appropriate. Risk of bias was judged using RoB 2 for randomized trials and ROBINS-I for non-randomized studies. Preclinical data (in vitro and animal studies) were not graded and were considered as mechanistic context only.

Following the database search, all identified records were imported into a citation management tool, and duplicates were removed. Full-text articles of potentially eligible studies were then retrieved and assessed in detail based on the inclusion and exclusion criteria. In total, 42 studies met all eligibility criteria and were included in the final review.

This methodology enabled the integration of diverse data sources while maintaining a structured and reproducible approach, in alignment with PRISMA standards for systematic reviews.

## 3. Results and Discussion

### 3.1. Metformin and Mitochondrial Function in ADPKD

In ADPKD, cystic renal epithelial cells exhibit profound mitochondrial dysfunction, with impaired oxidative phosphorylation and excess ROS production that drives cyst growth [11]. Metformin directly targets the mitochondria: it weakly inhibits respiratory complex I, reducing ATP production and raising the AMP/ATP ratio [12]. This energy stress activates AMPK, which in turn inhibits mTOR signaling and promotes mitochondrial biogenesis [11]. Indeed, in ADPKD models, metformin (often combined with glycolysis inhibitors) significantly lowers kidney Complex I activity and ATP levels, increasing AMPK phosphorylation, and slowing cyst expansion [13]. By dampening mitochondrial respiration and reducing oxidative stress, metformin may help re-balance energy metabolism in ADPKD renal cells [11,12].

### 3.2. Pathophysiology of ADPKD

ADPKD is a kidney disorder caused in majority of patients by mutations in the PKD1 or PKD2 genes, which encode polycystin-1 and polycystin-2, respectively. These proteins form a functional complex in the primary cilium of tubular epithelial cells and regulate intracellular calcium signaling, cell polarity, and mechanosensation. Disruption of polycystin function leads to reduced calcium influx, which in turn elevates intracellular cyclic AMP (cAMP) levels. Increased cAMP activates protein kinase A (PKA), driving proliferation of cyst-lining epithelial cells and stimulating CFTR-mediated chloride and fluid secretion, which contributes to cyst enlargement. Additionally, downstream signaling cascades such as B-Raf/MEK/ERK and mTOR are overactivated, promoting further cell growth and cystogenesis [14].

Another hallmark of ADPKD is loss of planar cell polarity, which leads to misoriented cell division and abnormal tubular architecture. Interstitial inflammation, epithelial–mesenchymal transition (EMT), and fibrosis accompany cyst expansion and accelerate the decline in kidney function. Emerging evidence also implicates dysregulated microRNAs (miRNAs) in ADPKD progression. Specific miRNAs (e.g., miR-21, miR-17) modulate gene networks involved in cell proliferation, apoptosis, and fibrosis, representing potential therapeutic targets [14].

Altogether, the pathogenesis of ADPKD involves a complex interplay of ciliary dysfunction, calcium and cAMP signaling disturbances, hyperproliferation, fluid secretion, inflammation, and fibrosis, leading to progressive cyst growth and loss of renal function over time.

### 3.3. Anti-Inflammatory and Molecular Mechanisms of Metformin in ADPKD

Metformin demonstrates significant anti-inflammatory activity, which may be of relevance in the pathophysiology of ADPKD. Chronic low-grade inflammation is increasingly recognized as a contributor to cyst growth, interstitial fibrosis, and disease progression. Metformin’s actions on several inflammatory mediators and intracellular signaling pathways may thus play a disease-modifying role in polycystic kidney disease.

At the molecular level, metformin activates AMPK, a central regulator of energy and redox homeostasis, which in turn suppresses the nuclear factor-κB (NF-κB) pathway—a master regulator of pro-inflammatory gene transcription [15,16]. Through inhibition of NF-κB signaling, metformin downregulates the expression of key cytokines such as tumor necrosis factor-α (TNF-α) and interleukin-6 (IL-6), both of which are implicated in renal injury, vascular dysfunction, and metabolic inflammation [15].

Notably, metformin also modulates the Toll-like receptor 4 (TLR4) cascade and reduces the activity of high mobility group box 1 (HMGB1), an alarmin that promotes inflammatory responses in damaged tissues. HMGB1, a validated direct binding target of metformin, acts as an endogenous ligand of TLR4 and can activate downstream inflammatory signaling including NF-κB and MAPK pathways. By directly suppressing HMGB1 release and binding, metformin interrupts this inflammatory loop [15].

Additional anti-inflammatory mechanisms of metformin involve attenuation of caspase-3 and caspase-8 activation—proteases involved in apoptosis and inflammation—as well as modulation of oxidative stress via nuclear factor erythroid 2-related factor 2 (NRF2) [15]. These pleiotropic effects suggest that metformin can influence both epithelial and immune pathways relevant to cyst growth and kidney remodeling in ADPKD.

Furthermore, the ability of metformin to suppress TGF-β signaling—a major fibrogenic and pro-inflammatory cytokine—provides another potential avenue for its antifibrotic and nephroprotective effects [15,17]. This mechanism could be particularly relevant in the context of ADPKD-associated interstitial fibrosis and CKD progression.

### 3.4. Effects on Carbohydrate Metabolism in ADPKD

ADPKD is increasingly recognized not only as a structural renal disorder but also as a condition marked by profound metabolic disturbances, particularly in carbohydrate metabolism [9]. A hallmark of metabolic reprogramming in ADPKD is a shift from oxidative phosphorylation to aerobic glycolysis, a phenomenon commonly observed in cancer cells. This shift supports rapid cyst-lining epithelial cell proliferation and excessive fluid secretion, thereby accelerating cystogenesis [18,19,20,21].

Furthermore, impaired insulin signaling and glucose intolerance are frequently observed in ADPKD patients, exacerbating systemic metabolic derangements. Metformin, a widely used antidiabetic drug, may counteract these abnormalities through its activation of AMP-activated protein kinase (AMPK) [22,23]. By targeting mitochondrial function, metformin reduces ATP production, thereby activating AMPK and promoting energy homeostasis [24]. This mechanism suppresses hepatic gluconeogenesis, enhances insulin sensitivity, and modulates glucose uptake and utilization [25]. These findings suggest that metformin may help slow cyst growth by targeting abnormal energy metabolism in ADPKD, in addition to improving glucose control.

### 3.5. Progression of Renal Failure in ADPKD

A substantial body of preclinical and clinical evidence suggests that metformin may attenuate the progression of renal dysfunction in ADPKD through modulation of key cystogenic and metabolic signaling pathways [26]. This aligns with the therapeutic priorities outlined in the KDIGO 2025 Clinical Practice Guideline, which emphasizes the importance of identifying and validating disease-modifying agents in the management of ADPKD [27]. As a pharmacological activator of AMPK, metformin exerts its effects via a downstream inhibition of the mechanistic target of rapamycin (mTOR) and the cystic fibrosis transmembrane conductance regulator (CFTR), both of which are essential mediators of cyst epithelial cell proliferation and chloride-driven fluid secretion, respectively [28,29,30]. In vitro and ex vivo studies conducted by Takiar et al. [31] demonstrated that AMPK activation via metformin induced significant cystic growth arrest through simultaneous inhibition of both CFTR and mTOR pathways [30,31]. These findings have been corroborated by in vivo models, where AMPK activation by metformin was associated with decreased cyst fluid accumulation and improved renal structural parameters [32]. Notably, in a Pkd1RC/RC mouse model, metformin reduced kidney-to-body weight ratios and cystic index, while preserving glomerular filtration rate (GFR) and reducing blood urea nitrogen (BUN) levels [33]. Similar renoprotective effects were observed in zebrafish models of polycystin-2 deficiency, where metformin modulated defective cellular processes, such as dysregulated autophagy and proliferation and decreased leukocyte infiltration in the pronephric region, suggesting an additional anti-inflammatory mechanism [34].

In translational and clinical settings, the efficacy and safety of metformin have been evaluated in several studies. A phase II double-blinded randomized placebo-controlled trial conducted by Seliger et al. [35] over 26 months in ADPKD patients with preserved renal function (eGFR ≥ 50 mL/min/1.73 m^2^) demonstrated that metformin slowed the decline of renal function relative to placebo [35]. These results were supported by a single-arm pilot study involving 34 non-diabetic ADPKD patients across CKD stages 1 to 5, which confirmed that metformin use was not associated with significant changes in eGFR or body mass index during follow-up, thereby reinforcing its safety even in advanced CKD [36]. Furthermore, in a retrospective analysis by Pisani et al., the comparison of diabetic ADPKD patients treated with metformin against matched non-diabetic controls revealed a lower overall risk of GFR loss in the metformin group, with linear mixed model analyses confirming the statistical significance of this trend [37].

Importantly, meta-analytical data support these observations. In a 2022 network meta-analysis, metformin was found to preserve GFR with a standardized mean difference (SMD) of 0.28 (−0.05; 0.61) compared to placebo, indicating a trend toward renoprotection, although not reaching statistical significance (*p* = 0.09) [5]. For comparison, tolvaptan demonstrated a statistically significant effect, with an SMD of 0.24 (0.16; 0.31), *p* < 0.001 [5].

Recent Cochrane analyses further explored metformin’s effects in randomized controlled trials. In pooled data from Brosnahan et al. and the TAME-PKD 2018 study (n = 71), the mean difference in change in eGFR between metformin and placebo was 2.82 mL/min/1.73 m^2^ (95% CI −0.29 to 5.92; *p* = 0.08), suggesting a non-significant but favorable trend [38]. Similarly, metformin was associated with slower rates of eGFR decline (MD = 2.94; 95% CI −1.93 to 7.81) and a reduced annual rate of eGFR loss (MD = 1.36; 95% CI −0.70 to 3.42), although these findings did not reach statistical significance [38]. Notably, no cases of kidney failure were reported in either the metformin or placebo group in the TAME-PKD trial, precluding estimation of relative risk for this endpoint [38].

Quantitative data from pooled analyses and subgroup analyses further suggest a potential benefit of metformin in slowing renal function decline. In one experimental cohort, the mean difference in eGFR decline between metformin-treated and control patients was 2.31 mL/min/1.73 m^2^ (95% CI: 0.82–3.79; *p* = 0.002) [7]. Another analysis reported an eGFR preservation of 3.05 mL/min/1.73 m^2^ in the metformin group versus controls [37,39]. In the TAME-PKD trial, metformin slowed the annual decline in eGFR by an estimated 1.37 mL/min/1.73 m^2^/year, although the difference did not reach statistical significance. However, a trend favoring slower height-adjusted total kidney volume (htTKV) growth was observed, particularly in subgroups with a baseline htTKV ≥ 800 mL/m or Mayo imaging classes 1C–E, suggesting metformin may be more effective in patients with rapidly progressive diseases [2,40].

In addition to monotherapy, metformin has demonstrated additive effects when used in combination with other agents targeting metabolic pathways. Co-administration of metformin with 2-deoxyglucose (2-DG) in ADPKD miniature pigs markedly reduced renal cyst formation and improved renal function by influencing enzymes involved in glycolysis and cellular proliferation [13]. Zhao et al. further demonstrated that such a combination therapy downregulated pro-proliferative signaling cascades including protein kinase A (PKA), extracellular signal-regulated kinase (ERK), and mTOR, while upregulating PI3K/Akt, thereby enhancing the therapeutic impact. Moreover, drug delivery innovations—such as kidney-targeting micelles (KMs) loaded with metformin and rapamycin—have enhanced tissue-specific efficacy and minimized systemic toxicity in experimental models, offering a promising future direction for precision treatment [28].

The cumulative evidence supports metformin as a potentially effective and safe disease-modifying agent in ADPKD. Its mechanism positions metformin as a promising adjunct or alternative to current therapies, such as tolvaptan, especially in individuals with metabolic alterations or rapidly progressing cyst burden. Continued investigation through large-scale randomized controlled trials will be essential to confirm its long-term impact on renal survival and to define the optimal patient population for targeted intervention.

### 3.6. Mortality in ADPKD: Potential Impact of Metformin

Observational studies have shown that metformin use is associated with reduced all-cause mortality in patients with stage G3 CKD, as demonstrated by Roussel [41] et al. in a cohort of over 5000 individuals, while no such benefit was observed in patients with more advanced CKD (stages G4–5). Similar patterns were reported in analyses from the Swedish National Diabetes Register and among U.S. veterans, where mortality risk was reduced in stage G3a but not in G3b CKD [42,43]. These findings are particularly relevant to the ADPKD population, as cardiovascular complications remain a leading cause of death. Recent evidence also suggests that metformin may be safer in CKD than previously thought and could reduce the risk of death and cardiovascular events, particularly in individuals with stage G3 CKD [44]. Its additional ability to improve insulin sensitivity, reduce inflammation, and ameliorate dyslipidemia may further support long-term survival in ADPKD, given the growing recognition of metabolic disturbances in this disease.

Preclinical studies have further supported this, with metformin administration in Pkd1-deficient mice showing no elevation in mortality despite some model-dependent adverse effects on renal morphology [45]. Importantly, clinical trials in ADPKD such as TAME-PKD and pilot studies involving both diabetic and non-diabetic patients reported no metformin-related mortality events, supporting its tolerability [2,24,40]. Nonetheless, the high rate of loss to follow-up and limited longitudinal data on hard endpoints in ADPKD cohorts remain challenges to definitive conclusions. Taken together, while the direct impact of metformin on mortality in ADPKD remains unproven, its cardiovascular and metabolic benefits suggest the potential to improve overall survival indirectly. Future large-scale, long-term trials are needed to assess mortality and other hard endpoints as primary outcomes in ADPKD patients receiving metformin.

A summary of the most relevant preclinical and clinical studies evaluating the effects of metformin in ADPKD is presented in Table 1, including information on study models, dosing, endpoints, and main findings.

### 3.7. Potential Variations in Metformin Efficacy by Sex, CKD Stage, and Diabetes Status

While metformin has shown promise as a disease-modifying therapy in ADPKD, emerging evidence suggests that its efficacy and safety may vary depending on the individual patient’s characteristics, including sex, CKD stage, and presence of diabetes.

Data on sex-specific responses to metformin in ADPKD are limited. Although not yet directly demonstrated in ADPKD, prior pharmacokinetic and endocrinological studies suggest that sex-related hormonal differences may influence metformin’s activity, particularly through modulation of AMPK signaling [46,47].

The stage of kidney disease appears to influence both the efficacy and tolerability of metformin. In patients with early-stage CKD (G1–G3a), metformin has been associated with a slower decline in eGFR, as reported in studies by Seliger et al. [35] and Brosnahan et al. [39]. In more advanced stages (G3b–G4), although current guidelines permit metformin use down to an eGFR of 30 mL/min/1.73 m^2^, concerns about MALA remain prevalent. Notably, available clinical data—including trials involving patients with CKD stages 4 and 5—have not shown increased adverse event rates. However, metformin’s effectiveness may be reduced in advanced disease due to altered pharmacodynamics and increased lactate accumulation [25,45].

Type 2 diabetes is an important modifier of ADPKD progression and may enhance metformin’s therapeutic impact. Diabetic patients often exhibit upregulation of glucose- and insulin-sensitive pathways, potentially amplifying metformin’s effects. In a retrospective analysis by Pisani et al. [37], diabetic ADPKD patients treated with metformin experienced a slower eGFR decline compared to non-diabetic controls. Similarly, a large cohort study by Roussel et al. [41] reported reduced all-cause mortality in patients with stage G3 CKD and diabetes treated with metformin, compared to those using other glucose-lowering agents.

### 3.8. Comparative Section: Metformin Versus Tolvaptan

Tolvaptan, a selective vasopressin V2 receptor antagonist, remains the only approved disease-modifying treatment for ADPKD with proven efficacy in slowing disease progression. However, its use is frequently limited by adverse effects, particularly polyuria. In a randomized, double-blind, placebo-controlled crossover study, Kramers et al. [9] demonstrated that both hydrochlorothiazide and metformin significantly reduced tolvaptan-induced 24 h urine volume (from 6.9 L to 5.1 L and 5.4 L, respectively; *p* < 0.001). While hydrochlorothiazide also improved markers of kidney injury, glucose metabolism, and quality of life, metformin showed no effect on these secondary outcomes [9].

A secondary analysis of the TEMPO 3:4 and REPRISE phase III trials by Stanley et al. [48] assessed the safety and efficacy of concomitant metformin and tolvaptan therapy. Although metformin users represented a small subgroup, no significant difference in the annualized decline of eGFR or increase in TKV was observed between the metformin + tolvaptan group and the tolvaptan-only group. Importantly, no safety signals or increased adverse event rates were associated with metformin co-treatment, suggesting that this combination is well tolerated [48].

Beyond clinical trials, experimental data from rodent models also support the potential benefits of metformin. Efe et al. [49] demonstrated that metformin restores urine-concentrating ability in rats and vasopressin V2 receptor knockout mice with nephrogenic diabetes insipidus (NDI) induced by tolvaptan. Metformin activates AMPK, leading to increased phosphorylation and membrane accumulation of aquaporin-2 (AQP2) and urea transporter A1 (UT-A1), independently of the vasopressin signaling pathway. This mechanism resulted in increased urine osmolality and reduced polyuria, sustained over several days of treatment [49].

Collectively, these findings indicate that metformin may represent a safe adjunctive or alternative therapeutic strategy in ADPKD, particularly in patients who experience significant aquaretic side effects from tolvaptan or have concomitant diabetes. While tolvaptan remains the gold standard for direct disease modification, metformin may offer indirect renal benefits via modulation of metabolic pathways such as AMPK, mTOR, and CFTR. Ongoing phase III clinical trials (e.g., IMPEDE-PKD, NCT04939935 [50]) will further clarify metformin’s place in ADPKD treatment, including in combination with tolvaptan [48].

It should be emphasized that the therapeutic considerations presented in this review are based on preliminary studies and mechanistic reasoning rather than confirmed clinical trial evidence. To date, no large randomized controlled trials have demonstrated the efficacy of metformin in patients with ADPKD, and all proposed strategies should therefore be regarded as exploratory and subject to further validation. The therapeutic recommendations discussed here should be interpreted cautiously and primarily as hypothesis-generating. Ensuring a balanced description of various agents and strategies is also important, to avoid overemphasis on those with limited or heterogeneous evidence.

## 4. Discussion

This systematic review, conducted in accordance with the PRISMA guidelines, consolidates current evidence on the effects of metformin in ADPKD, highlighting its potential as a disease-modifying agent. The synthesis of data from both preclinical and clinical studies indicates that metformin, primarily via AMPK activation, may attenuate cyst growth, reduce fluid secretion, and preserve renal function [7,8]. Nevertheless, despite promising preclinical efficacy, the translation into consistent clinical benefit remains limited, largely due to the heterogeneity of ADPKD progression, variability in study designs, and the scarcity of large-scale RCTs [51]. KDIGO 2025 guidelines emphasizes the urgent need for high-quality clinical research to evaluate emerging therapies, including metabolic modulators such as metformin, and to support evidence-based treatment decisions in ADPKD [27].

This review encompasses a wide spectrum of evidence ranging from in vitro experiments and animal models to small clinical pilot trials and meta-analyses. Such diversity allows for a comprehensive overview of the topic but simultaneously limits the strength of the conclusions. Findings derived from animal and cellular studies are essential for elucidating molecular mechanisms; however, their translatability to the human population remains restricted. Similarly, small-scale clinical trials and pilot studies are prone to systematic bias and low statistical power, which may lead to overestimation of treatment effects. Moreover, the variability of outcome measures, including eGFR, TKV and metabolic biomarkers, hampers direct comparability and complicates synthesis across studies. Consequently, data interpretation should be undertaken with caution, and future research should aim to standardize methodology and harmonize clinical endpoints to improve reliability and reproducibility.

Our findings suggest several key directions for future research. First, stratified analysis and precision medicine approaches should be incorporated in upcoming RCTs to identify patient subgroups most likely to benefit from metformin. Evidence from included studies points toward a greater therapeutic impact in early-stage disease, whereas some later-stage models demonstrated neutral or even adverse effects, potentially due to lactate-driven activation of pro-cystogenic pathways [52]. These findings emphasize the importance of treatment timing and support future investigations into lactate metabolism and mitochondrial function in ADPKD pathophysiology.

Second, optimal dosing strategies and safety thresholds require further clarification. Although current guidelines permit metformin use in patients with an eGFR ≥ 30 mL/min/1.73 m^2^, fears of metformin-associated lactic acidosis (MALA) persist, particularly in nephrology. While the included studies consistently reported a favorable safety profile—even in patients with impaired renal function—metformin’s use in non-diabetic CKD, including ADPKD, lacks robust evidence from controlled clinical trials. Future research should include well-powered, blinded RCTs with predefined safety monitoring criteria, particularly in populations at higher risk for metabolic complications [25]. Notably, the ongoing IMPEDE-PKD trial (Trial registration number: NCT04939935) is designed to evaluate the safety and efficacy of metformin in ADPKD patients and may provide further insights into its potential as a disease-modifying therapy [50].

Third, the potential for combinatorial therapy deserves special attention. Several preclinical studies included in this review demonstrated synergistic effects when metformin was used. Moreover, novel kidney-targeted delivery systems (e.g., micelles, nanoparticles) have shown potential to enhance renal bioavailability and minimize systemic toxicity. These strategies should be prioritized in future translational research to improve therapeutic efficacy and safety [53].

An additional consideration relates to the safety and feasibility of metformin therapy in the ADPKD population. Metformin is generally well tolerated; however, the risk of lactic acidosis, particularly in patients with reduced renal function, remains a relevant concern. Common adverse effects such as gastrointestinal intolerance (e.g., nausea, diarrhea) may also limit adherence in some patients. Careful monitoring of renal function and metabolic parameters is therefore necessary, and dose adjustments may be required in advanced CKD or in patients undergoing dialysis. These factors highlight the need for well-designed clinical studies to determine the real-world safety and feasibility of metformin in ADPKD.

While this review was conducted using PRISMA methodology to ensure a comprehensive and unbiased synthesis of the current literature, several limitations must be acknowledged. First, the included studies were heterogeneous in design, patient population, metformin dosing, and outcome measures, limiting the ability to conduct meta-analytic pooling. Second, most clinical data were derived from small-scale studies or secondary analyses, and often lacked comparator arms, blinding, or long-term follow-up. Third, confounding factors such as concurrent use of the renin–angiotensin–aldosterone system (RAAS) inhibitors, differences in baseline kidney function, and genetic variability (e.g., PKD1 vs. PKD2) were often insufficiently controlled or reported. Heterogeneity of study populations represents an important limitation of the current evidence base. The included studies enrolled patients of different ages, disease stages, and comorbidities, such as type 2 diabetes, which may substantially influence treatment response and outcomes. This variability makes it challenging to generalize mechanistic conclusions across all individuals with ADPKD. For example, the potential benefits of metformin might be more pronounced in patients at earlier stages of the disease, when renal reserve is preserved, than in those with advanced nephropathy. Recognizing this heterogeneity is essential to avoid overgeneralization and highlights the need for future studies with stratified designs that account for disease stage and relevant comorbid conditions

## 5. Conclusions

This systematic review highlights the emerging role of metformin as a potential disease-modifying therapy in ADPKD, although large randomized controlled trials are still lacking. The novel contribution of this review lies in its integrated approach, combining mechanistic insights with clinical evidence and outlining new therapeutic strategies, including the potential of metformin as a complementary or alternative option to established treatments. Through AMPK activation, metformin may attenuate cyst growth, improve renal outcomes, modulate glucose metabolism, and exert anti-inflammatory effects. While preclinical and early clinical studies suggest a favorable impact on disease progression and systemic complications, the current evidence remains insufficient to support routine clinical use in ADPKD. High-quality, large-scale randomized controlled trials are necessary to validate these findings, determine optimal patient selection and dosing, and explore long-term effects on renal survival, mortality, and quality of life. Until such data become available, metformin should be considered a promising but investigational option in the therapeutic landscape of ADPKD.

## Figures and Tables

**Table 1:** Summary of preclinical and clinical studies evaluating the effects of metformin in ADPKD.

Study	Model/Participants	Endpoints	Key Findings
Takiar et al. [31]	In vitro, ex vivo	Cystic growth, CFTR/mTOR activity	AMPK activation via metformin inhibited cyst growth
Pastor-Soler et al. [33]	Pkd1RC/RC mice	Kidney/body weight ratio, cystic index, GFR, BUN	Reduced cystic index, improved renal function
Chang et al. [34]	Zebrafish, polycystin-2 deficiency	Cyst formation, autophagy, inflammation	Reduced cystogenesis and inflammation
Seliger et al. [35]	ADPKD patients (RCT)	eGFR decline	Slower eGFR decline vs. placebo
Sorohan et al. [36]	34 ADPKD patients (pilot)	eGFR, BMI	No significant change; confirmed safety
Pisani et al. [37]	Diabetic ADPKD patients vs. controls	GFR loss	Lower GFR loss in metformin group
Tsukamoto et al. [5]	Meta-analysis	GFR preservation	Trend toward benefit (SMD = 0.28, *p* = 0.09)
St Pierre et al. [38]	RCTs (Brosnahan, TAME-PKD)	eGFR change, failure	Trend toward slower eGFR decline
Yao et al. [7]	Meta-analysis	eGFR	eGFR benefit (MD = 2.31 mL/min/1.73 m^2^)
Lian et al. [13]	Miniature pigs	Cyst formation, renal function	Synergistic effect, improved renal function
Kramers et al. [9]	ADPKD patients	Urine volume, QOL	Reduced polyuria with tolvaptan
